# GCS 15: when mild TBI isn’t so mild

**DOI:** 10.1186/s42466-018-0001-1

**Published:** 2019-02-28

**Authors:** Latha Ganti, Tej Stead, Yasamin Daneshvar, Aakash N. Bodhit, Christa Pulvino, Sarah W. Ayala, Keith R. Peters

**Affiliations:** 1 UCF HCA Emergency Medicine Residency Program of Greater Orlando, University of Central Florida College of Medicine, Orlando, FL USA; 2 Polk County Fire Rescue, University of Central Florida, Orlando, FL USA; 3 University of Central Florida, Orlando, USA; 4 New York College of Podiatric Medicine, New York, NY USA; 5 Department of Neurology, Saint Louis University, Saint Louis, MO USA; 6 Department of Emergency Medicine, University of Cincinnati, Cincinnati, OH USA; 7 Touro College of Osteopathic Medicine, Mare Island, CA USA; 8 Division of Neuroradiology, University of Florida, Gainesville, FL USA

## Abstract

**Objective:**

The present study characterizes patients with the mildest of mild traumatic brain injury (TBI), as defined by a Glasgow coma score (GCS) of 15.

**Methods:**

This is an IRB approved observational cohort study of adult patients who presented to the emergency department of a Level-1 trauma center, with the primary diagnosis of TBI and a GCS score of 15 on arrival. Data collected included demographic variables such as age, gender, race, mechanisms of injury, signs and symptoms including associated vomiting, seizures, loss of consciousness (LOC), alteration of consciousness (AOC), and post-traumatic amnesia (PTA).Pre- hospital GCS, Emergency Department (ED) GCS, and results of brain CT scans were also collected as well as patient centered outcomes including hospital or intensive care unit (ICU) admission, neurosurgical intervention, and in hospital death. Data were stored in REDCap (Research Electronic Data Capture), a secure, web- based application. Descriptive and inferential analysis was done using JMP 14.0 for the Mac.

**Results:**

Univariate predictors of hospital admission included LOC, AOC, and PTA, all *p* < 0.0001. Patients admitted to ICU were significantly more likely to be on an antiplatelet or anticoagulant (*P* < 0.0001), have experienced PTA (*p* = 0.0025), LOC (*p* < 0.0001), or have an abnormal brain CT (p < 0.0001). Patients who died in the hospital were significantly more likely to be on an antiplatelet or anticoagulant (*P* = 0.0005. All who died in the hospital had intracranial hemorrhage on ED head CT, despite having presented to the ED with GCS of 15. Patients were also significantly more likely to have had vomiting (p < 0.0001). Patients who underwent neurosurgical intervention were significantly more likely to be male (*P* = 0.0203), to be on an antiplatelet or anticoagulant (P = < 0.0001) likely to have suffered their TBI from a fall (*P* = 0.0349), and experienced vomiting afterwards (*P* = 0.0193).

**Conclusions:**

This study underscores: 1) the importance of neuroimaging in all patients with TBI, including those with a GCS 15. Fully 10% of our cohort was not imaged. Extrapolating, these would represent 2.5% bleeds, and 1.47% fractures. 2) The limitations of GCS in classifying TBI, as patients with even the mildest of mild TBI have a high frequency of gross CT abnormalities.

## Introduction

Traumatic brain injury (TBI) accounts for over 1 million US emergency department visits annually [1], 275,000 civilian hospitalizations [2] and 21,000 military [3] injuries. Traumatic brain injury can have lasting consequences with neurocognitive deficits [4–7], post concussive symptoms [8–10], and repeat return visits to the emergency department [11].

Traumatic brain injury has traditionally been classified as mild, moderate and severe based on the Glasgow Coma Scale (GCS), a scoring system never intended to classify brain injury per se but rather level of consciousness. Developed originally in 1974 [12] then modified in 1976 [13], the GCS consists of eye opening, motor, and verbal components, for a total of 15 points. A TBI consensus workgroup points out that while the GCS can be useful in the clinical management and prognosis of TBI, it “does not provide specific information about the pathophysiologic mechanisms which are responsible for the neurologic deficits and targeted by interventions [14].”

Nonetheless, this score is still widely used today. While a GCS of 13 to 15 is considered mild traumatic brain injury (mTBI) per the American College of Rehabilitation Medicine [15], studies have shown that an mTBI with GCS 13 is not the same as one with GCS 15 [16]. Indeed, even in patients who have a GCS of 15, the mTBI is not always benign. This study characterizes those patients with the mildest of mTBI, as defined by a GCS of 15, and describes the acute injury features, as well as clinical outcomes.

## Methods

This study is derived from a subset of data from a previously published cohort study [17] which included adult patients who presented to the emergency department (ED) of a Level-1 trauma center, over an 18-month period with the primary complaint of TBI (ascertained using ICD-9 codes of 800–804.9, 850–854, and 959.01). For the current study, the patients had to have a GCS of 15 upon ED arrival, and the injury had to have occurred within 24 h prior to presentation. Demographic variables such as age, gender, past medical history, and medications were abstracted, in addition to the mechanism of injury, and associated signs and symptoms such as vomiting, seizures, loss of consciousness (LOC), alteration of consciousness (AOC), and post- traumatic amnesia (PTA). The patient was considered to have AOC if the neurologic exam revealed a decreased mental status, or if the patient reported feeling dazed or confused or having difficulty thinking. Varibales collected are summarized in the Table 6 in Appendix.

The prehospital and emergency department (ED) GCS were also recorded, as well as vital signs and computed tomography (CT) scan results. This study was approved by our Institutional Review Board. Data were stored in Research Electronic Data Capture (REDCap), a secure web-based application. Descriptive and inferential data analysis was done using JMP 14.0 for Mac.

## Results

The cohort (*n* = 2211) was 57% male. The marital status was 61% single, 27% married, 7% divorced or separated, and 6% unknown. The median age was 37 years (IQR = 23–57), with a range of 18–101 years. Cohort demographics are summarized in Table 1.

**Table 1:** cohort demographics

age	median = 37 years; IQR = 23–57; range 18–101.
gender	57% male
race	83% white; 14% black; 3% hispanic
marital status	61% single, 27% married, 7% divorced or separated, 6% unknown
taking an anti-thrombotic agent?	11% on some agent. 3% warfarin; 7% aspirin; 3% clopidogrel; < 1% each of heparin, low molecular weight heparin, aspirin-dipyridamole combination
mechanism of injury	fall 48%, motor vehicle collision 34%, assault 30%
came via ambulance?	62%

**Fig. 1:**
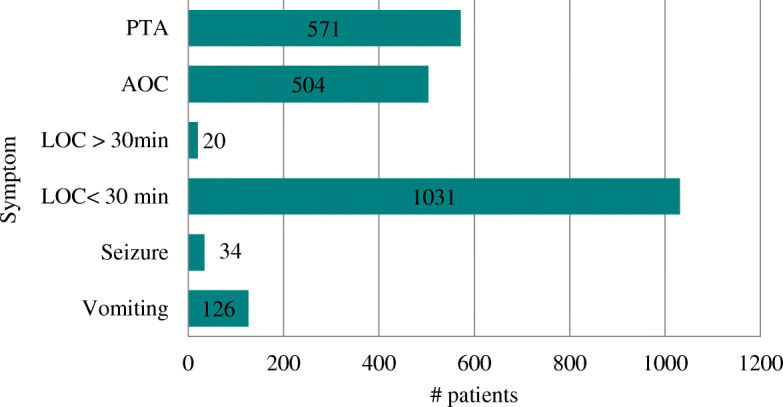
Frequency of symptoms

**Fig. 2:**
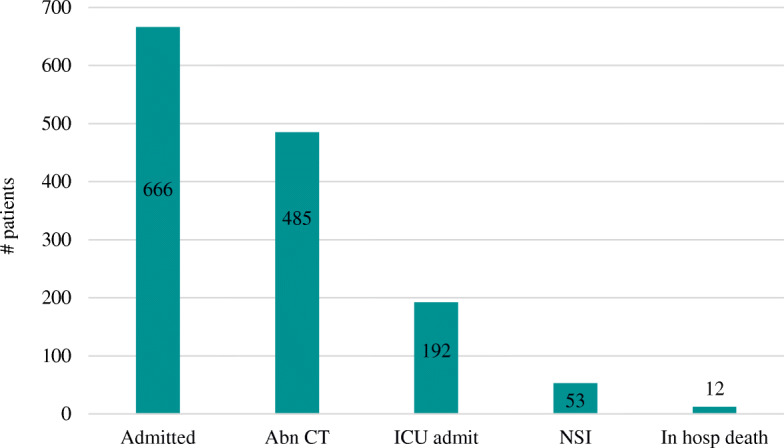
Frequency of Clinical Outcomes

The most common symptomatology associated with a GCS of 15 was LOC followed by PTA and AOC. The most common mechanism of injury was fall at 48%, followed by motor vehicle collision at 34%, and assault or being struck in the head at 30%. The most common location of injury reported was on the road (39%), followed by inside a home (21%). The frequency of symptoms is summarized in Fig. 1, and clinical outcomes are summarized in Fig. 2.

More than half the cohort (55%) reported no alcohol in the 12 h preceding the head injury, while 17% admitted to drinking, and another 27% were “unknown”. Alcohol levels were obtained in only 233 patients, or 10% of the cohort. The range was from 0 to 441, with a median of 153, and an IQR of 37–241. Interestingly, a higher alcohol level was significantly associated with *not* being admitted to the hospital (*P* < 0.0001), and did not result in any higher association with having an abnormal head CT either.

The prehospital GCS was obtained for all 946 patients transported via ambulance. It ranged from 3 to 15, with a median of 15 and an interquartile range of 15–15. Thus, the majority of the patients had a prehospital GCS of 15. However, 3% had a prehospital GCS of 13, and 17% had a 14. A total of 838 patients were brought in by ambulance, while 86 were brought in by helicopter. More people brought in by helicopter (48%) vs ambulance (21%) had a prehospital GCS < 15 (P < 0.0001). Regardless, having a prehospital GCS < 15 was significantly associated with being admitted to the hospital (*P* < 0.001) and having an abnormal CT scan (P < 0.001).

A third of the cohort (30%) were admitted to the hospital, of which 192 (29%) were admitted to the Intensive Care Unit (ICU). Univariate predictors of hospital admission included LOC, AOC, and PTA, all *p* < 0.0001. In multivariate analysis, only LOC and PTA retained statistical significance. Having a lower prehospital GCS was also significantly associated with being admitted to the hospital (*p* < 0.0001, 95% CI -0.0677 to − 0.1523). However, none of the symptoms or prehospital GCS were significantly associated with ICU admission.

Compared to the general population, patients admitted to the ICU (Table 2) were significantly older, with a median age of 62 (IQR = 41–78). Patients admitted to the ICU were significantly more likely to be on an antiplatelet or anticoagulant agent (*p* < 0.0001, 95%CI -0.3153 to − 0.4047) with warfarin, aspirin and clopidogrel retaining independent statistical significance. Patients admitted to the ICU were also significantly more likely to have experienced PTA (*p* = 0.0025, 95% CI -0.0352 to − 0.1648), LOC (*p* < 0.0001, 95% CI -0.3282 to − 0.4318) or have an abnormal brain CT (p < 0.0001, 95% CI -0.6584 to − 0.7816).

**Table 2:** Factors associated with ICU admission

	Age	Gender	War-farin	ASA	Pla-vix	Any AC	Fall	MVC	PH GCS < 15	Head CT	LOC	Vomit	PTA	AOC
Total cohort minus ICU *N* = 2019	Median 35, range 18–101, IQR 23–54	57% male	0, 0%	106, 5%	40, 2%	146, 7%	939 47%	689 34%	160/946 17%	315, 14%	212, 11%	115, 6%	504, 25%	153 8%
ICU *N* = 192	Median 62, IQR 41–78, range 18–95	59% male	16, 8%	42, 22%	25, 13%	83 43%	122, 64%	57, 30%		170 88%	95 49%	13 7%	67, 35%	65 3%

Patients who died in the hospital (Table 3) were significantly more likely to be on an antiplatelet or anticoagulant agent: warfarin (*P* = 0.0051, 95% CI -0.042 to − 0.238), aspirin (*p* = 0.0005, 95% CI -0.1139 to − 0.4061), or clopidogrel (p < 0.0001, 95% CI -0.1214 to − 0.3186). All patients in the hospital death group had intracranial hemorrhage on ED head CT, despite having presented to the ED with a GCS of 15, and all but one patient also having a pre-hospital GCS of 15. Patients were also significantly more likely to have presented with vomiting (p < 0.0001, 95% CI -0.5514 to − 0.8286) and were older with a median age of 81, compared to the survivor cohort median age of 37.

**Table 3:** Factors associated with in-hospital death

	Age	Gender	Warfarin	ASA	Plavix	Any AC	Fall	MVC	PH GCS < 15	Head CT	LOC	Vomit	PTA	AOC
Total cohort minus in hosp death *N* = 2199	Median 37, IQR 23–56, range 18–101	57% male	59 3%	144, 7%	62, 3%	146 7%	1052 48%	744 34%	189 9%	1984, 90%	102,447%	125 6%	568, 26%	503 23%
Death *N* = 12	Median 81, IQR 70–85, range 18–92	50% male	2, 17%	4 33%	3 25%	9 75%	9 75%	2 17%	1 8%	12 100%	7 58%	3 75%	3 25%	1 8%

Patients who underwent neurosurgical intervention (Table 4) were significantly more likely to be male (*P* = 0.0203), to be on an antiplatelet or anticoagulant agent: warfarin (P = < 0.0001), aspirin (*P* < 0.0001), or clopidogrel (*P* = .0003). They were also more likely to have suffered their TBI from a fall (*P* = 0.0349), and experienced vomiting afterwards (*P* = 0.0193). Fourteen patients had their anticoagulant status reversed. 11 received IV vitamin K, 7 received both IV vitamin K and fresh frozen plasma (FFP), 2 received FFP only, and 1 received SC vitamin K.

**Table 4:** Factors associated with neurosurgical intervention

	Age	Gender	Warfarin	ASA	Plavix	Any AC	Fall	MVC	ph GCS < 15	Head CT	LOC	Vomit	PTA	AOC
Total cohort minus surgery *N* = 2158	Median 36, IQR 23–56, range 18–101	56% male	52, 2%	137, 6%	59, 3%	220, 10%	1028, 48%	742, 34%	200/917, 22%	1944, 90%	1016, 47%	121, 6%	561, 26%	491, 23%
Surgery *N* = 53	Median 64, IQR 41–78, range 20–86	72% male	9, 17%	11, 21%	6, 11%	21, 40%	33, 62%	4, 8%	8/20, 40%	52, 98%	15, 28%	7, 13%	10, 19%	13, 25%

**Fig. 3:**
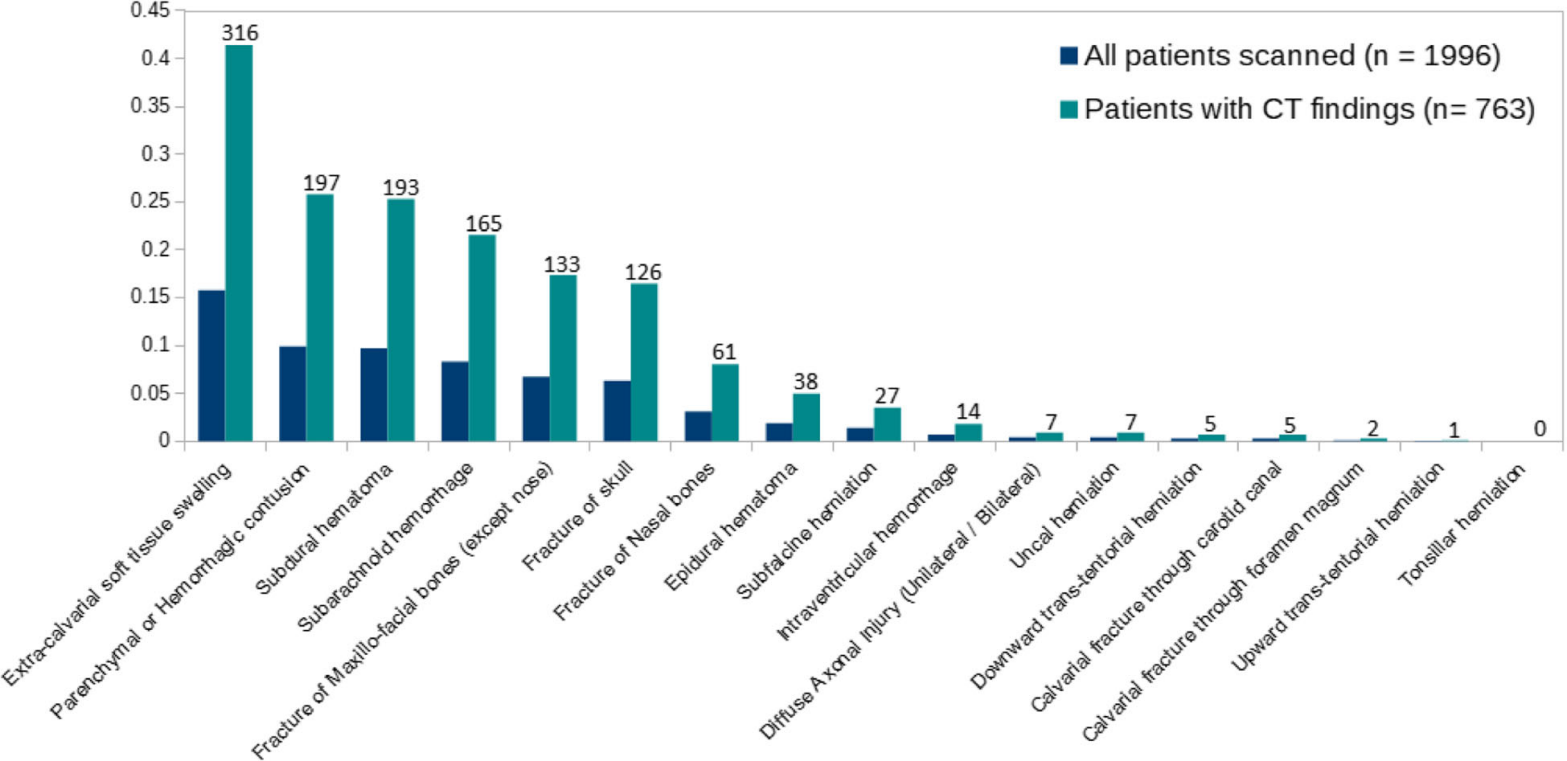
Proportion of CT findings

A total of 1996 or 90% of patients had a brain CT scan. Of these, 485 or 24% had a CT abnormality (Table 5). The frequency of specific CT abnormalities is summarized in Fig. 3. The most common CT lesions noted on CT were extra-calvarial soft tissue swelling (41%), parenchymal or hemorrhagic contusion (26%), subdural hematoma (25%), and subarachnoid hemorrhage (22%). Patients who had an abnormal CT scan were significantly more likely to be older, on an anticoagulant, and have suffered a fall as their TBI mechanism (all *P* < 0.001).

**Table 5:** Factors associated with an abnormal CT scan

	Age	Gender	Warfarin med = 0	ASA med = 1	Plavix Med = 2	Any AC	Fall	MVC	ph GCS < 15	LOC	Vomit	PTA	AOC
CT normal *n* = 1511	Median 33, IQR 22–51, range 18–95	57% male	30, 2%	52, 3%	17, 1%	95, 6%	678, 45%	545, 36%	127/639, 20%	752, 50%	88, 6%	405, 27%	297/1365, 22%
CT abnormal *n* = 485	Median 59, IQR 37–77, range 18–101	63% male	31, 6%	85, 18%	45, 9%	134, 28%	307, 63%	119, 25%	84/248, 34%	245, 51%	34, 7%	152, 31%	151/481, 31%

## Discussion

In this observational study of emergency department presentations for patients with a GCS of 15 upon arrival, a number of findings emerge that suggest that the simple characterization of head injury by GCS score may not be sufficient.

The cohort is somewhat unique in the proportion of patients who received a brain CT as part of their evaluation. Indeed, there are several rules that specifically seek to limit the use of head CT for head injury, within certain parameters. The Canadian head CT [18] rule excludes people on blood thinners, and those who had a seizure after the head injury. Also, a number of high-risk criteria have been noted with the rule, including use of blood thinners, any suspicion of skull fracture, age > 65, and a change in GCS level. Medium risk criteria including a “dangerous mechanism” is also noted as a caveat. The New Orleans head CT criteria [19] actually targets only the GCS 15 head injury population, and “suggests patients with GCS 15 and head trauma are unlikely to need a head CT as long as they do not have headache, vomiting alcohol or drug intoxication, persistent anterograde amnesia, seizure, visible trauma above the clavicles.”

Clearly, neither of these rules are optimal for the mild TBI cohort in this emergency department study. Studies published years after these rules concur, suggesting that “Patients with GCS 15 and risk factors or neurological symptoms should be evaluated with CT scan, [as] the outcome of mild TBI depends on the combination of preinjury, injury and postinjury factors” [20]. CT abnormalities are in fact not uncommon in mild TBI. A study of 2766 patients with mild TBI imaged in ED found that every sixth patient (16.1%) with mild TBI had an intracranial lesion [21]. The most common lesions were subdural hematomas, subarachnoid hemorrhages, and contusions. Similar to the current study, the authors noted that a lower Glasgow Coma Scale score, male sex, older age, falls, and chronic alcohol abuse were associated with higher risk of acute intracranial lesion in patients with mild TBI. These findings underscore the heterogeneity of neuropathology associated with the mild TBI classification.

Even in cases of mTBI, patients sometimes require neurosurgical intervention (NSI), as noted in the current study. In their study of mTBI patients, Tierney et al. [22] also noted that 8.2% had some form of NSI performed (including placement of an intracranial pressure monitor and other measuring devices). The in-hospital death rate for the NSI group was 13%, significantly higher than general figures for mTBI ranging from 0.3–1.8%. The use of anticoagulants as well as age being over 60 predicted a bad neurological outcome.

ICU admission for mTBI, while seemingly counterintuitive, is not all that uncommon. A retrospective study of 595,171 mTBI patients in the National Trauma Data Bank found that 44.7% were admitted to an ICU, While 17.3% of these met the criteria for overtriage, as defined by: ICU stay ≤ 1 day; hospital stay ≤ 2 days; no intubation; no neurosurgery; and discharged to home [23], that still leaves 27.4% of mTBI patients who were deemed appropriate for ICU admission. Interestingly, the study noted that a common “risk factor” for overtriage included isolated subarachnoid hemorrhage.

## Conclusion

This study underscores: 1) the importance of considering neuroimaging in all patients with TBI, including those with a GCS 15. In the current study, a total of 10% of the cohort was not imaged. If the CT data is extrapolated to the non-imaged group, the proportion of bleeds and fractures would be increased by 2.5 and 1.5 percentage points, respectively. 2) recognizing the limitations of GCS in classifying TBI, as patients with even the mildest of mild TBI have a high frequency of gross CT abnormalities, and non-benign outcomes including ICU admission, neurosurgical interventions and even in-hospital death.

## Appendix

**Table 6:** Study variables

	Variable / Field Name	Field choices	N complete (out of 2211 unless noted otherwise)
ed_arrivel_date	ED Arrival Date	date	2211
age_in_years	Age	number in years	2211
sex	Gender	0, Female | 1, Male	2211
race	Race	0, Caucasian | 1, Black | 2, Hispanic | 3, Native American | 4, Asian | 5, Other	2211
marital_status	Marital Status	0, Single | 1, Married | 2, Divorced/ Separated | 3, Not known	2210
employment	Employment Status	0, Not employed | 1, Employed | 2, Retired | 3, Student | 4, Not known	2656
date_head_injury	Date of Head Injury	date	1959
time_head_injury	Time of Head Injury	0, 0–12 h ago | 1, 12–24 h ago | 2, More than 24 h ago	2192
head_injury_reporting	Head Injury Reporting	0, Self Reported | 1, Friend/ family | 2, EMS	2211
asso_vomit	Vomiting after injury	0, No | 1, Yes	2210
asso_seizure	Seizures after injury	0, No | 1, Yes	2209
loss_consciousness	Loss of Consciousness	0, No | 1, Yes | 2, Unknown	2211
aoc	Altered Mental Status/Alteration of Consciousness (AOC)	0, No | 1, Yes | 2, Unknown	2211
pta	Post Traumatic Amnesia (PTA)	0, No | 1, Yes | 2, Unknown	2211
fall	Association of Fall with Injury	0, No | 1, Yes | 2, Unknown	2211
occupational	Was this an occupational injury?	0, No | 1, Yes | 2, Unknown	2210
hit_struck_fall_head	Did anything hit/struck/fall on head?	0, No | 1, Yes | 2, Unknown	2211
sport	Did the injury occur while playing a sport?	0, No | 1, Yes | 2, Unknown	2211
recreational_activity	Injury occurred during a recreational activity?	0, No | 1, Yes | 2, Unknown	2210
rec_vehicle	Was a recreational vehicle involved?	0, No | 1, Yes | 2, Unknown	379/379
rec_vehicle_type	What type of Rec Vehicle was involved?	0, Bicycle | 1, Motorcycle | 2, ATV | 3, Other | 4, Watercraft | 5, Not Known	290/290
helmet_use	Use of Helmet in Recreational Vehicle injury	0, No | 1, Yes | 2, N/A	284/290
traffic_accident	Did the Injury occur in a traffic accident?	0, No | 1, Yes | 2, Unknown	2211
traffic_accident_driver	Location of Patient	0, Driver | 1, Front Seat Passenger | 2, Back Seat Passenger | 3, Open Air Seating | 4, Other | 5, Unknown	746
seatbelt	Was a seatbelt worn?	0, No | 1, Yes | 2, N/A	742
alcohol_bf_injury	Alcohol Consumption Prior to Injury (by Patient)	0, No | 1, Yes | 2, Unknown	2211
assaulted	Was the patient Assaulted?	0, No | 1, Yes | 2, Unknown	2211
location_injury_occured	At what location did the injury occur?	0, Inside a Home | 1, Outside a home (yard/driveway) | 2, On the road | 3, “Outdoors” | 4, At Work | 5, Restaurant or Bar/Club | 6, Store | 7, Other Public Area (Park/sidewalk) | 8, Other	2211
transfer	Was the patient transferred from another facility?	0, No | 1, Yes	2211
mode_trans	Mode of Transportation	0, Walk in/private vehicle | 1, Ambulance | 2, Helicopter	2211
admitted_dis_or	Discharged or Admitted	0, Discharged | 1, Admitted	2211
hlos	Hospital Length of Stay	number value	666/666
icu	ICU Stay?	0, No | 1, Yes	666/666
icu_los	ICU Length of Stay	number value	192/192
disch_disposition	Discharge Disposition	1, routine dc| 2, rehab | 3, psych | 4, expired| 5, hospice/NH| 6, AMA| 7, law enforcement	2211
in_hospital_death	In hospital death?	0, No | 1, Yes	2211
prehosp_gcs_completion	Pre Hospital Glasgow Coma Scale Completion	0, No | 1, Yes	946/1381
	Pre Hospital GCS	number from 3 to 15	
tbi_severity_prehosp	TBI Severity- Pre Hospital	0, Mild | 1, Moderate | 2, Severe	946/1381
ed_gcs_completion	ED Glasgow Coma Scale Completion	0, No | 1, Yes	2211
ed_gcs_score_1	ED GCS Score	number from 3 to 15	2211
tbi_severity_ed	TBI Severity- ED	0, Mild | 1, Moderate | 2, Severe	2211
surgical_intervention	Surgical intervention done?	0, No | 1, Yes	2211
ct_scan	CT Scan Done?	0, No | 1, Yes	2211
head_ct_abmormal	Head CT abnormal?	0, No | 1, Yes	1996/1996
ct_findings	Please check any/all abnormalities that are observed on the CT scan:	0, Extra-calvarial soft tissue swelling | 1, Fracture of skull | 2, Fracture of Maxillo-facial bones (except nose) | 3, Fracture of Nasal bones | 4, Calvarial fracture through carotid canal | 5, Calvarial fracture through foramen magnum | 6, Subfalcine herniation | 7, Upward trans-tentorial herniation | 8, Downward trans-tentorial herniation | 9, Uncal herniation | 10, Tonsillar herniation | 11, Epidural hematoma | 12, Subdural hematoma | 13, Subarachnoid hemorrhage | 14, Intraventricular hemorrhage | 15, Parenchymal or Hemorrhagic contusion | 16, Diffuse Axonal Injury (Unilateral / Bilateral)	485/485
alcohol_level	Alcohol Blood Level	number value	233
urine_drug_screen	Urine Drug Screen	0, Negative | 1, Positive	157
arr_meds	Arrival Anticoagulant Medications?	0, No | 1, Yes	2167
med_type	Medications- Types	0, Warafrin | 1, Aspirin | 2, Plavix | 3, Aggrenox | 4, Ticlid | 5, Dipyridamole | 6, Heparin | 7, Enoxaparin | 8, Dabigatran	2167/2167
anticoag_rev_int	Anticoagulation Reversal Intervention?	0, No | 1, Yes	189/242
int_type	Intervention- Type	0, IV Vitamin K | 1, FFP | 2, PCC | 3, Factor IX Complex | 4, Recombinant Factor VIIa | 5, SC Vitamin K | 6, Other	14/14

## Abbreviations

AOC: Alteration of consciousness
CT: Computer tomography
ED: Emergency department
GCS: Glasgow coma score
ICU: Intensive care unit
LOC: Loss of consciousness
PTA: Post-traumatic amnesia
RedCap: Research Electronic Data Capture
TBI: Traumatic brain injury
